# Nanotopography Induced Human Bone Marrow *Mesangiogenic Progenitor Cells* (MPCs) to *Mesenchymal Stromal Cells* (MSCs) Transition

**DOI:** 10.3389/fcell.2016.00144

**Published:** 2016-12-20

**Authors:** Sara Antonini, Marina Montali, Emanuela Jacchetti, Sandro Meucci, Paolo D. Parchi, Serena Barachini, Francesca M. Panvini, Simone Pacini, Iacopo Petrini, Marco Cecchini

**Affiliations:** ^1^NEST, Istituto Nanoscienze-CNR and Scuola Normale SuperiorePisa, Italy; ^2^Department of Clinical and Experimental Medicine, University of PisaPisa, Italy; ^3^Dipartimento di Chimica, Materiali e Ingegneria Chimica “G.Natta”, Politecnico di MilanoMilan, Italy; ^4^Department of Surgical, Medical and Molecular Pathology and Critical Care Medicine, University of PisaPisa, Italy; ^5^Department of Translational Research and New Technologies in Medicine and Surgery, University of PisaPisa, Italy

**Keywords:** mesangiogenic progenitor cells, mesenchymal stromal cells, bone marrow culture, polyethylene terephthalate, nanograting

## Abstract

*Mesangiogenic progenitor cells* (MPCs) are a very peculiar population of cells present in the human adult bone marrow, only recently discovered and characterized. Owing to their differentiation potential, MPCs can be considered progenitors for mesenchymal stromal cells (MSCs), and for this reason they potentially represent a promising cell population to apply for skeletal tissue regeneration applications. Here, we evaluate the effects of surface nanotopography on MPCs, considering the possibility that this specific physical stimulus alone can trigger MPC differentiation toward the mesenchymal lineage. In particular, we exploit nanogratings to deliver a mechanical, directional stimulus by contact interaction to promote cell morphological polarization and stretching. Following this interaction, we study the MPC-MSC transition by i. analyzing the change in cell morphotype by immunostaining of the key cell-adhesion structures and confocal fluorescence microscopy, and ii. quantifying the expression of cell-phenotype characterizing markers by flow cytometry. We demonstrate that the MPC mesengenic differentiation can be induced by the solely interaction with the NGs, in absence of any other external, chemical stimulus. This aspect is of particular interest in the case of multipotent progenitors as MPCs that, retaining both mesengenic and angiogenic potential, possess a high clinical appeal.

## Introduction

*Mesenchymal stromal cells* (MSCs) have been the object of extensive research for decades, due to their intrinsic clinical value. *Mesangiogenic progenitor cells* (MPCs), instead, were only recently discovered and characterized. They were firstly co-isolated, in different percentages, with MSCs in human adult bone marrow cultures applying autologous serum (Petrini et al., 2009); later, the establishment of specific culture conditions selective for MPCs allowed the isolation of these cells with a high grade of purity (>95%). Specifically, when human bone marrow mononuclear cells (hBM-MNCs) are cultured in basal medium supplemented with human serum (HS) on hydrophobic surfaces, it is possible to select slow-cycling MPCs after 6 days of culture (Trombi et al., 2009) because this was the only cell population present in the initial preparation capable of attaching on hydrophobic plastic dishes.

Phenotypically, MPCs are easily distinguishable from MSCs for their fried egg-shape morphology and peculiar immuophenotype characterized by the positivity to CD18, CD31 and nestin as well as negativity to the MSC markers as CD73 and CD90. Interestingly, as MPCs initially showed to retain mesengenic, cardiomyogenic, and angiogenic potential, these cells were firstly named “*mesodermal*” progenitor cells. Later on, the mesengenic potential was definitively demonstrated and finely described in two steps of differentiation, once cultured in specific pre-formulated media for MSC expansion. Namely, MPCs firstly differentiate into slow cycling CD90^+^/nestin^+^ cells (*early*MSCs), activating non-canonical Wnt signaling, that generate exponentially growing MSC-like cells (*late*MSCs) after prolonged culture time (Fazzi et al., 2011). Similarly, angiogenic potential of MPCs was demonstrated. Also in this case the process involves two steps: first, MPCs are induced to VEGF-stimulated angiogenic sprouting; then they complete differentiation by forming capillary tube-like structures in 3D-cultures. Conversely, the cardiomyogenic potential was not definitively demonstrated, suggesting changing the MPC nomenclature into “*mesangiogenic* progenitor cells” (Montali et al., 2016) in spite of “*mesodermal*” progenitor cells. MPCs have been demonstrated deriving from a unique bone marrow cell population named *Pop#8* (Pacini et al., 2016). This cell population has been sorted from adult human bone marrow as CD45^low^CD31^bright^CD64^bright^CD14^neg^ and showed similarities to monocytoid progenitors. Moreover, MPC morphology and phenotype partially resemble macrophages suggesting that these mesangiogenic progenitors and their *in vivo* counterparts (*Pop#8*) could belong to the hemopoietic compartment. Similarly, plasticity of peripheral monocytes and macrophages has been demonstrated. Some groups reported evidences showing that these cells could differentiate into fibroblast-like and collagen-producing cells called “fibrocytes” which participate in tissue repair, mainly sustaining fibrosis (reviewed in Bucala, 2015). Nonetheless, demonstration of genuine mesengenic potential (osteogenic, adipogenic, and chondrogenic) of fibrocytes is still lacking, restricting their tissue repairing potential to scar formation, while sustaining to angiogenesis has been reported for these cells but only as paracrine secretion of pro-angiogenic factors (Grieb et al., 2011). Moreover, fibrocytes derived from peripheral CD45^bright^CD14^+^ monocytes also characterized for the expression of CD11b, C11c, CD13, and CD16 (Pilling et al., 2009), which have not been detected on bone marrow-derived CD45^low^CD14^neg^ *Pop#8*.

As MPCs can be considered progenitors for MSCs, they potentially represent a promising cell population to apply for skeletal tissue regeneration applications. Cell based medicinal products obtained by hBM-MNCs cultured in autologous serum were applied in the treatment of upper limb nonunions (Giannotti et al., 2013). In this paper, authors reported consistent percentages of MPCs (1–10%) within the expected MSC population, although not all of the conditions for MPC selection were complained. Nonetheless, it was suggested that the MPC fraction possibly contributed to the long-term healing reported in the treated patients. This hypothesis arose from the reported MPC plasticity suggesting that, once implanted, these cells could support osteogenesis differentiating into *early*MSCs, as well as contributing to the neo-vascularization of the engineered construct thank to their angiogenic potential.

In order to better investigate and exploit this possible therapeutic scenario in the field of orthopedics, further experiments should be performed *in vitro* in order to predict the MPC differentiation fate resulting from different physico-chemical stimulations that selectively mimic specific aspects of the *in vivo* micro-environments.

Bone tissue homeostasis represents a complex biological process finely regulated by humoral stimuli as hormones, growth factors and cytokines as well as by cell-cell and cell-matrix contact interactions (Florencio-Silva et al., 2015). Thus, optimal bone regenerative therapy should enhance mineralized tissue healing through enrichment of the bone defect with a micro/nanostructured matrix scaffold to support the wound, with cells that will give raise to osteoprogenitors and proper biochemical stimuli. Recently, a factor controlling the fate of many osteocompentent cells has been introduced, taking in consideration that micro- e nano-topography of the bone architectures could have a role in the regulation of the activity of the bone cells trough the activation of cellular mechanotransduction mechanisms mediated by adhesion molecules (Green et al., 1995; Zohar, 2012). In particular, this complex bone architecture resulted mainly sustained by collagen and hydroxyapatite (HA), which together form a highly aligned composite matrix that contribute to the toughness and strength of bone itself (Weiner et al., 1999; Kerschnitzki et al., 2011). Collagen triple helices are typically around 300 nm long and 1.5 nm in diameter (Weiner et al., 1999) conferring a linear topography to the bone structure at the nanoscale. Many studies reported the influence of nanotopography to the biology of osteoprogenitors (Dalby et al., 2007; McMurray et al., 2011; Janson et al., 2014) suggesting to control their differentiation applying nanostructured surfaces of orthopedic implants.

MPCs showed particular adhesion properties sustained by podosome-like structures, that were applied for the definition of a MPC selective culture method (Trombi et al., 2009). Further studies demonstrated that gelsolin-served F-actin podosomial structures were re-organizedn in paxillin-served F-actin stress fibers, during the mesengenic differentiation of MPCs (Pacini et al., 2013), suggesting that topographical stimuli could play a crucial role in the MPC fate.

The aim of this study is evaluating the effects of surface nanostructuring on MPCs, considering the possibility that the nanotopography alone can trigger the MPC differentiation toward the mesenchymal lineage. In particular, we exploited nanogratings to deliver a mechanical, directional stimulus by contact interaction to promote cell morphological polarization and stretching. Following this interaction, we studied the MPC-MSC transition by (i) analyzing the change in cell morphotype by immunostaining of the key cell-adhesion structures and confocal fluorescence microscopy, and (ii) quantifying the expression of cell-phenotype characterizing markers by flow cytometry.

## Materials and methods

### Nanograting fabrication

NGs were fabricated by thermal nanoimprinting lithography (NIL) on copolymer 2-norbornene ethylene cyclic olefin copolymer (COC) foils (IBIDI, Martinsried, Germany). COC was chosen because of its well-documented biocompatibility and optimal optical properties for high-resolution fluorescence microscopy. NIL is based on the combination of pressure and heat, which aids the transfer of the chosen pattern from a rigid mold to thermoplastic materials. Molds were fabricated by electron beam lithography (EBL) and reactive ion etching (RIE) techniques as previously reported (Cecchini et al., 2007). COC foils were imprinted using an Obducat Nanoimprint 24 system (Obducat, Sweden). After cleaning with nitrogen flow, the substrates were placed on top of the silicon molds and softened by raising the temperature up to 150°C. A pressure of 50 bar was then applied for 5 min before cooling down to 70°C, that is below the glass transition temperature of the copolymer (Tg = 134°C). Finally, the pressure was released and the mold detached from the substrate with a scalpel. The imprinted substrates were quality checked by optical microscopy and attached to the bottom of hollowed 35 mm Petri dishes by using silicone glue (RS Components RS692–524).

### Donors and sample collection

Human bone marrow blood samples were collected, after written consent, from 12 patients (5 Male/6 Female, median age 64) during orthopedic surgery for hip replacement. The study has been performed according to the declaration of Helsinki and to the approval of the local ethical committee of “*Azienda Ospedaliero-Universitaria Pisana*.” A 20 ml syringe containing 500 U.I. of heparin was used to aspirate 10 ml of fresh tissue immediately after femoral neck osteotomy and before femoral reaming. Samples were promptly sent to the cell culture facility and processed soon after.

### MPC preparation and characterization

#### Cell preparation

MPCs were obtained from hBM applying selective culture conditions, according to the previously reported method (Trombi et al., 2009; Montali et al., 2016). Briefly, bone marrow blood samples were diluted 1:4, carefully stratified on Ficoll-Paque™ Premium (GE Healthcare, Uppsala, Sweden) and centrifuge at 400 g for 30′. hBM mononuclear cells (hBM-MNCs) were collected at the interface of the density gradient, washed in Dulbecco's modified phosphate saline buffer (D-PBS, Thermo Scientific, Carlsbad, USA-CA) and resuspended in Dulbecco's modified Eagle medium (DMEM, Thermo Scientific) supplemented with 10% of pooled human type AB serum (PhABS, Lonza, Basel, Switzerland), 1:100 Glutamax™ (Thermo Scientific) and 100 μU/ml of penicillin and streptomycin (Thermo Scientific). Cell concentration and vitality were determined by Bürker hemocytometer. From 30 × 10^6^ to 60 × 10^6^ hBM-MNCs were then plated in a T75 culture flask for suspension cultures (Greiner Bio-one, Monroe, USA-NC) and incubated at 37°C and 5% CO_2_ for 48 h in DMEM/10%PhABS. Cells in suspension were removed with the medium change and then adherent cell were maintained in culture for 6 days. At the end, medium was discard, flasks were washed with D-PBS and 1,5 ml of TrypLE® Select detaching solution (Thermo Scientific) was applied for 10′ to collect MPCs.

#### Flow cytometry

3 × 10^5^ freshly isolated MPCs were processed for immunophenotyping and incubated with anti-CD11c VioBlue®-conjugated, anti-CD18 PE-conjugated, anti-CD31 PE/Cy7-conjugated and anti-CD90 APC-conjugated (all from Miltenyi Biotec, Bergisch Gladbach, Germany) for 30′ at 4°C in the dark. Cells were then washed with MACSQuant® Running Buffer (Miltenyi Biotec) and resuspended in 500 μl of the same buffer for data acquisition in MACSQuant® Flow Cytometer (Miltenyi Biotec). Data were acquired and analyzed by MACSQuantify® analysis software (Miltenyi Biotec). Samples with percentage of CD11c^+^CD18^+^CD31^+^CD90^neg^ lower than 95% were excluded from the study.

#### Nestin detection and F-actin organization

From 8 × 10^5^ to 1.6 × 10^6^ hBM-MNCs were seeded in 2-well culture chamber slides (Thermo Scientific) and cultured in DMEM/10%PhABS for 6 days, as described above. Cultures were then washed twice in D-PBS and MPCs were fixed in paraformaldehyde 4% for 15′. Permeabilization was performed by incubation in Triton X-100 0.5% (Sigma Aldrich) for 15′, after the removal of the fixative by extensive wash in D-PBS. Slides were blocked applying Image-iT™ FX signal enhancer (Thermo Scientific) and incubated with anti- human nestin monoclonal antibody (Abcam, Cambridge, UK) overnight. Antibody excess was removed by washing in Triton X-100 0.5% and slides were then incubated with AlexaFluor® 488-conjugated secondary antibody for 1 h. F-actin staining was performed by AlexaFluor® 555-conjugated phalloidin (Thermo Scientific) for 20′. The slides were finally mounted with ProLong® anti-fade reagent with DAPI and pictures were taken using an inverted fluorescence DM IRB microscope (Leica, Wetzlar, Germany) equipped with LAS AF image analysis software (Leica).

#### Mesengenic differentiation by pre-formulated medium

Freshly detached MPCs were seeded in T75 flasks (20,000 cells/cm^2^) and let adhere overnight in DMEM/10%PhABS. The day after the medium was changed with MesenPRO® RS medium (Thermo Scientific) and the cells were cultured until confluence (P1-MSCs). Cells were then detached by enzymatic digestion with TrypLE® Select and sub-cultured at 5000 cells/cm in new T75 flasks for flow cytometry analysis, and in 2-well culture chamber slides for the detection of nestin (performed at confluence, P2-MSCs).

### MPC culture on T2 nanogratings

Freshly isolated MPCs were resuspended and adjusted to 40,000 cells/ml in DMEM/10%PhABS, then a cell suspension volume of 500 μl was seeded into four T2 nanostructured inserts, in a drop. Cells were let adhere for 4 h, then additional fresh medium (1.5 ml) was added and cells were cultured for 7 days. In parallel, cultures have been performed on four “FLAT” inserts; additional cultures were also prepared in standard 6-wells plates for suspension cultures, in order to evaluate the spontaneous cell differentiation (marked as “CTRL”). For flow cytometry quantification of the mesengenic differentiation, the cells cultured onto the three different substrates were detached by TrypLE® Select digestion solution, washed in *MACS*Quant® Running Buffer (MiltenyiBiotec), and incubated with the monoclonal antibodies, as described above. The acquisition and analysis were performed applying dot-plot template set up for MPC/P2-MSCs analysis. Mesenchymal differentiation was evaluated by calculating the ratio between events with MSC-associated phenotype, detected in *R2* gate, and MPC-related events detected in *R1* gate (MSC/MPC).

### Statistical analysis

Data were reported as average value ± the standard error of the mean (mean ± SEM), obtained from at least three independent experiments. Data were statistically analyzed by GraphPad PRISM 6.00 program (GraphPad Software, San Diego, CA, USA). For parametric data, Student's *t*-test (unpaired, two-tailed) or One-Way ANOVA (Tukey's or Dunnett's multiple comparison test) analysis were used; the mean values obtained in each repeated experiment were assumed to be normally distributed about the true mean. Statistical significance refers to results where *p* < 0.05 was obtained. Further details on data representation and statistics are reported in the figure legends.

## Results

### Nanograting fabrication and characterization

**Figure 1:**
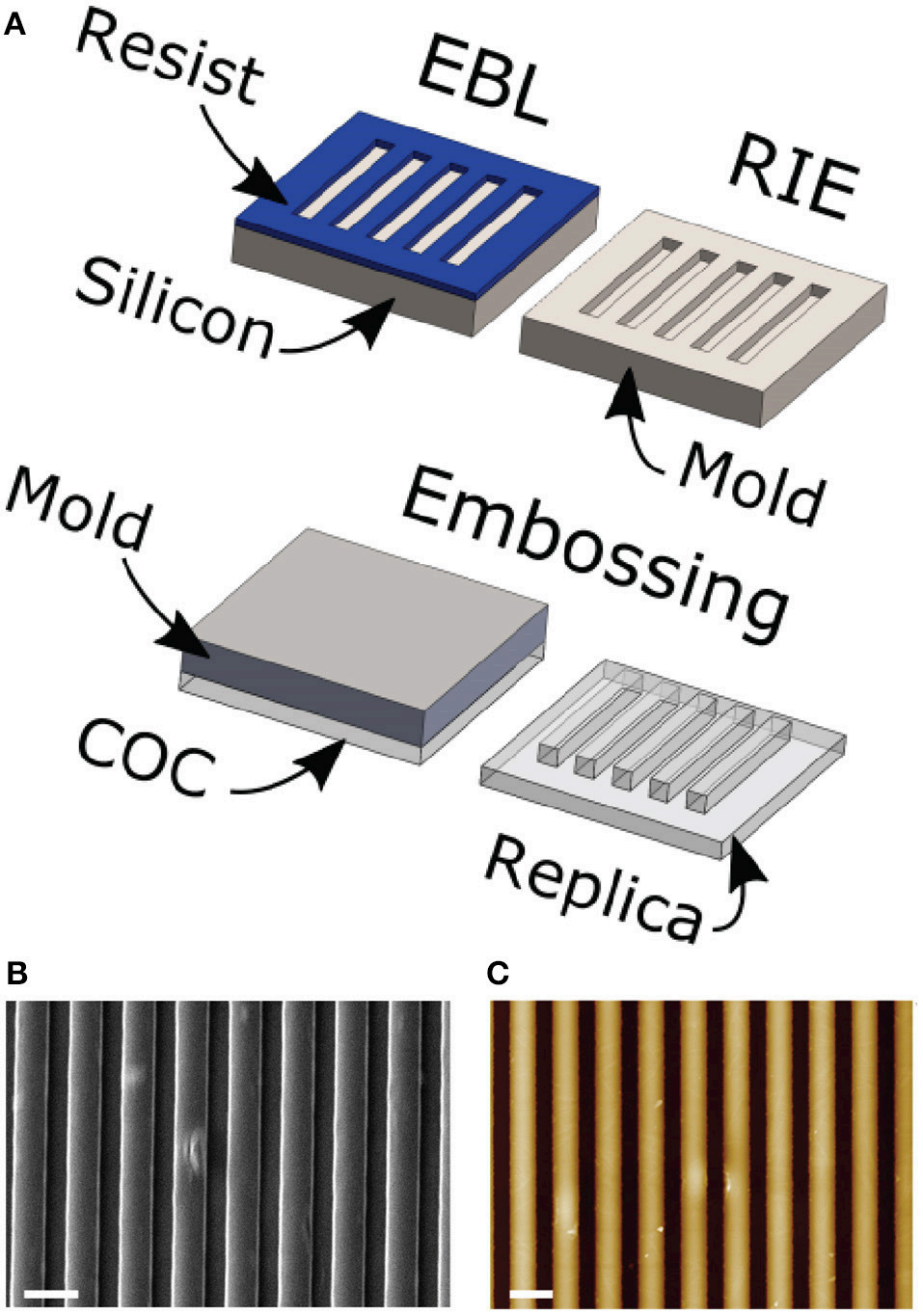
**(A)** Scheme of the substrate fabrication process. COC foils are placed on nano/microstructured molds and imprinted following a single heating and pressure cycle. Scanning electron microscope **(B)** and atomic force microscope **(C)** representative images of a T2 substrate. Scale bars = 2 um; The color-scale in **(C)** goes from 0 (black) to 400 nm (white).

Three different substrates were chosen to study MPC differentiation toward the MSC phenotype. The substrates were produced starting form 200-μm-thick COC films by hot embossing (Figure 1), as detailed in the Materials and Methods section. This process provided us with nanostructured surfaces covering macroscopic areas (= 1 cm^2^), well suitable for fluorescence microscopy and cell/molecular biology studies. Two surface geometries were fabricated, the first having an embossed nanograting (named T2) while the second was obtained with the same thermal and pressure cycle but using a flat mold (i.e., a silicon polished wafer). This last substrate is named FLAT and has been used as control condition for the T2 surface in each experiment. T2 is characterized by ridge and groove width of 1000 nm, and depth of 350 nm. All the COC surfaces were proven to be adhesive for MPCs without requiring any chemical functionalization but plasma activation. In this study and previously (Tonazzini et al., 2013; Jacchetti et al., 2014) we did not find any cytotoxicity originating by either the surface topographical modification or by the material itself. Finally, standard tissue culture Petri dishes were also used as control condition for the FLAT substrates in order to verify that the material itself did not cause the MPC-MSC transition. Summarizing, three different substrates were exploited for this study, the T2 and FLAT (both in COC), and a standard plastic Petri dish (named CTRL).

### MPC culture characterization

**Figure 2:**
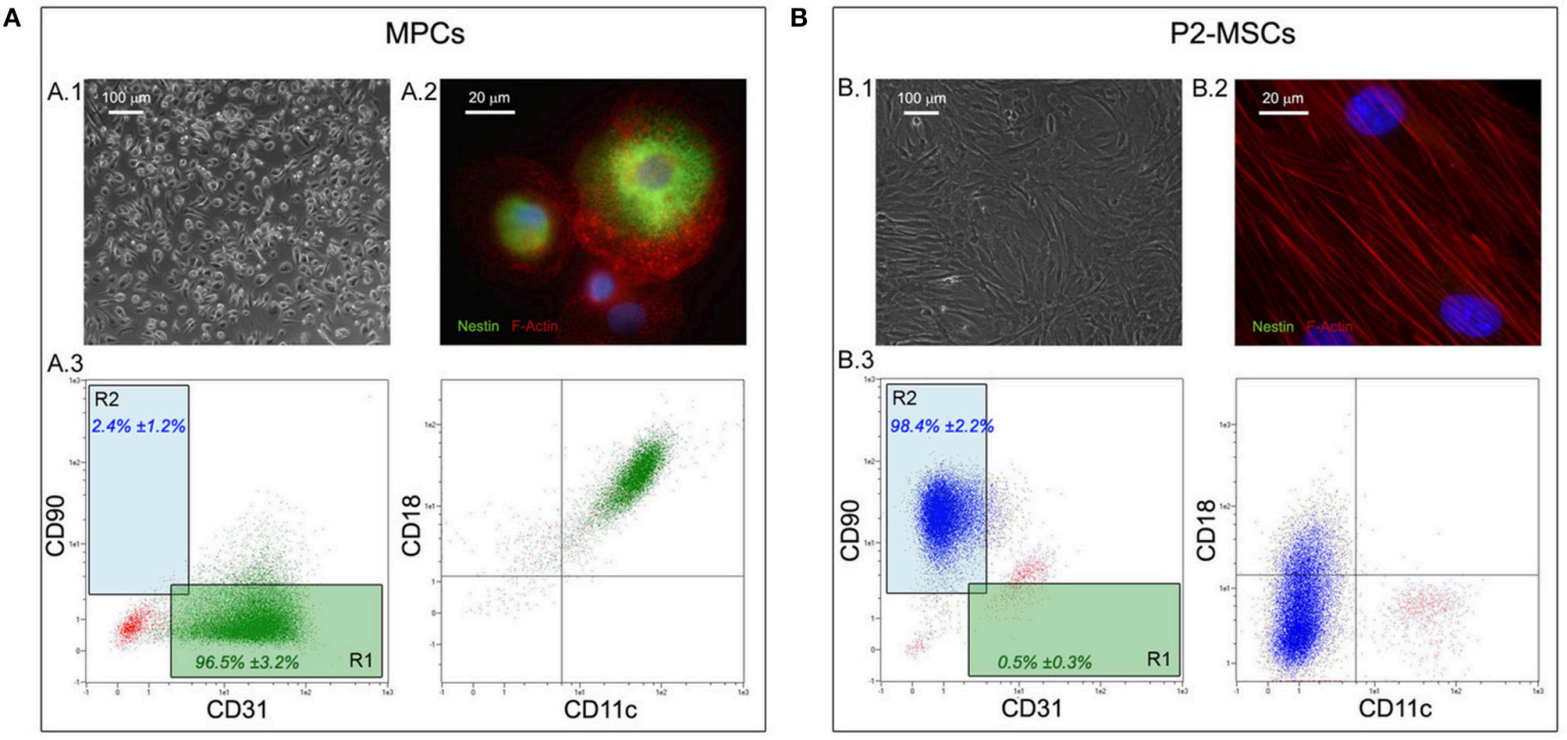
**Isolation and characterization of hBM-derived MPCs**. Culturing hBM-MNCs for 6 days under MPC selective conditions lead to almost homogenous population of rounded and highly rifrangent adherent cells **(A.1)**. These cells were definitively characterized as MPCs for their intense positivity to nestin (green in **A.2**) and for the presence of numerous podosome-like structures characterized by the dotted distribution of F-Actin (red in **A.2**). Flow cytometry confirmed the MPC phenotype on approximately all analyzed cells **(A.3)**, which were displayed in the lower right quadrant of the CD90 vs. CD31 dot-plot (“R1” green box). After two passages under mesengenic differentiating conditions (P2-MSCs) the cultures were constituted by proliferating and fibroblastoid MSC-like cells **(B.1)** and nestin was no longer detected **(B.2)**. All MPC-related markers resulted undetectable by flow cytometry on P2-MSCs **(B.3)**, which expressed high levels of CD90, occupying the upper left quadrant of the CD90 vs. CD31 dot-plot (“R2” blue box).

All of the bone marrow samples could generate MPCs (Figure 2A), that were isolated by applying the previously reported method (Trombi et al., 2009; Montali et al., 2016). As expected, culturing hBM-MNCs for 6 days under selective culture conditions led to monomorphic cultures of adherent cells with a peculiar fried egg-shape morphology (Figure 2A.1). These cells were then characterized as MPCs for their intense expression of nestin (green in Figure 2A.2) and the typical dotted distribution of F-actin, that reveals numerous podosome-like structures (red in Figure 2A.2). Also flow cytometry confirmed the expected immunophenotype characterized by the positivity to CD31 (PECAM), CD18 (Integrin β2), CD11c (Integrin αX) and the lack of CD90 expression (Figure 2A.3). Flow cytometry was then applied to determine the purity of MPC cultures before subculture the cells on NGs. Most of the analyzed cell preparations revealed MPC percentages higher than 95% (mean 96.5 ± 3.2%, *n* = 10), determined applying the *R1* gate on CD90 vs. CD31 dot-plot (green box in Figure 2A.3), and were then processed for subsequent culture. Otherwise, two MPC primary cultures revealed purity lower than 95% (data not shown) with a consistent percentage (10 and 8%) of MSC-like cells, determined applying the *R2* gate (blue box in Figure 2A.3). These latest two cell preparations were excluded from the study.

### Specific formulated media induced differentiation of MPCs

In order to verify the differentiation potential of the isolated MPCs, two passages of mesengenic differentiation were applied. After 2 weeks of culture in MesenPRO® RS medium, differentiated cells (P2-MSCs) showed a fibroblastoid and spindle-shaped morphology, typical of MSC-like cells (Figure 2B.1). F-actin resulted re-organized in stress-fibers (red in Figure 2B.2), while very low levels of nestin expression was reported in few rare cells. Flow cytometry revealed almost homogeneous culture of CD90-positive cells, lacking MPC-related markers as CD31, CD18, and CD11c (Figure 2B.3). As a consequence, in the CD90 vs. CD31 dot-plot these cell events were plotted in the *R2* gate. In order to definitely confirm the MSC nature of the differentiated cells, P2-MSC cultures were exposed to adipogenic as well as osteogenic differentiating conditions, and respectively the lipid droplet accumulation and calcium deposition were report for the cultures investigated (*n* = 10, data not shown).

### MPCs interact with NGs

**Figure 3:**
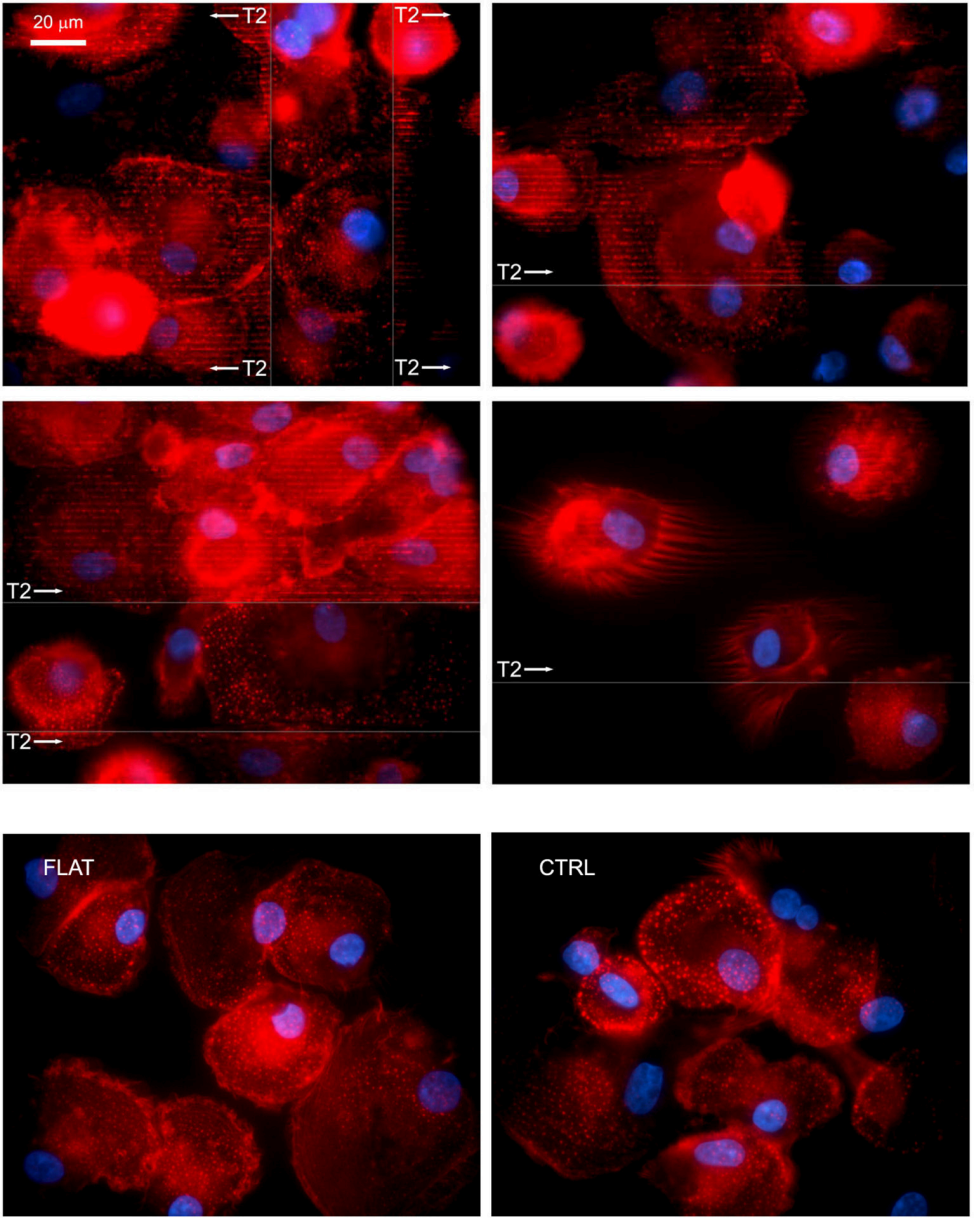
**MPCs interact with NGs at the adhesion level**. After overnight incubation on nano-structured T2 gratings, most of the seeded MPCs resulted firmly attached and showed the numerous podosomes aligned to the grating direction (white arrows) and in correspondence to the crests. Similarly, filopodia showed an alignment to the grating direction. Fluorescence images were acquired by confocal microscopy and immunostaining of F-Actin; arrows show grating direction, and area. The bottom row shows cells on FLAT (left) and CTRL (right).

Initial cell attachment on T2 nanostructures was not significantly altered. After the 4 h incubation, most of the seeded cells resulted firmly adherent to the substrate surfaces without any noticeable difference with respect to those on FLAT or CTRL. After overnight incubation, MPC morphology resulted highly conserved, with cells maintaining the typical rounded fried egg-shape. Nuclei appeared slightly oval with compact chromatin, and did not show any alignment to the grating direction. Conversely, all characteristic podosome-like structures resulted aligned in correspondence to the NG crests, while resulting randomly oriented in correspondence of the not patterned areas, similarly to those of the cells seeded on FLAT or CTRL (Figure 3). Moreover, numerous filopodia, occasionally detected on some MPCs, showed the ability to align to the NGs.

### Nanogratings trigger MPC differentiation toward MSC-like cells

**Figure 4:**
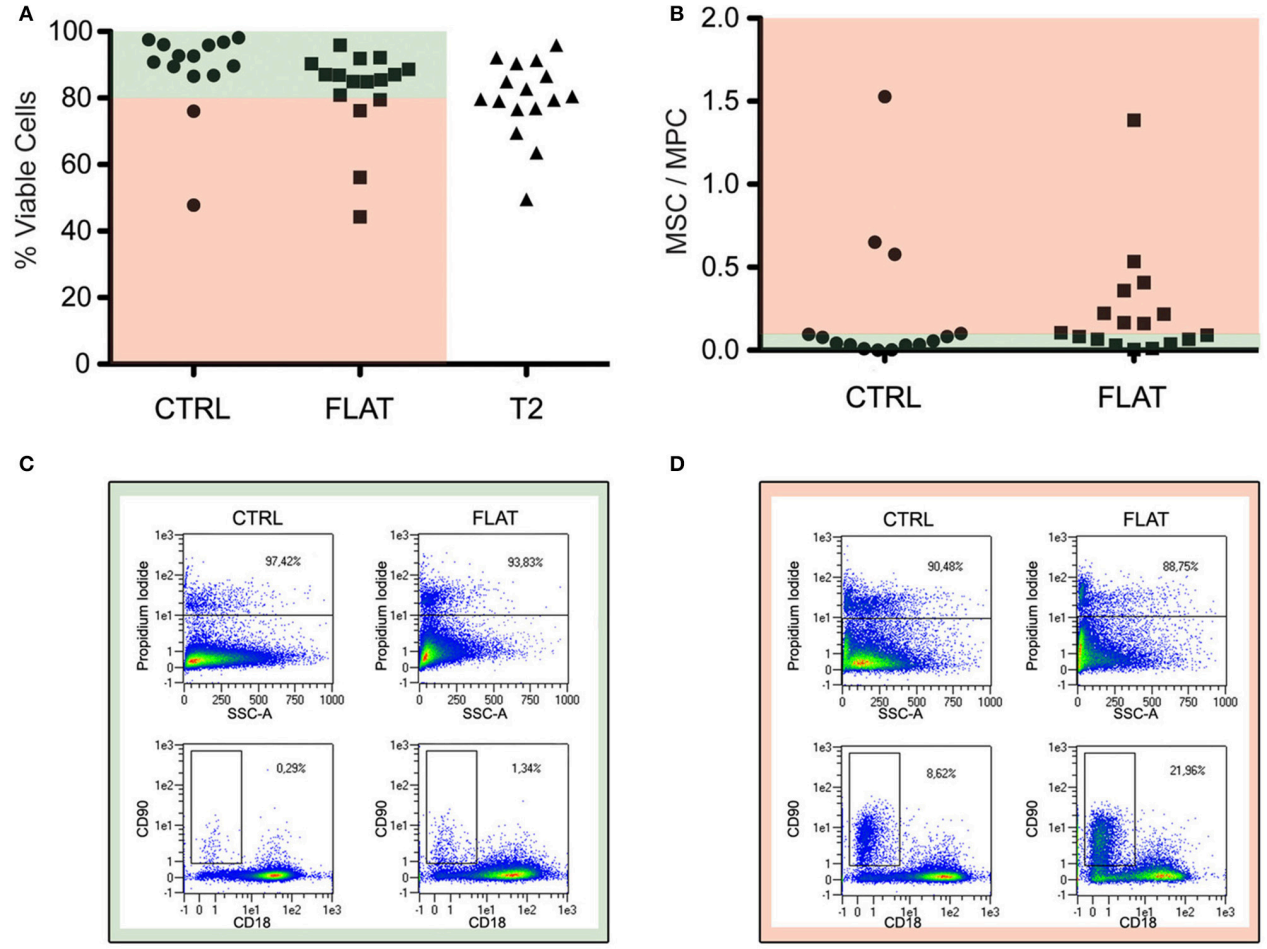
**Evaluation of cell viability and spontaneous differentiation. (A)** Percentage of viable cells (PI-negative) for the different substrates. **(B)** Spontaneous differentiation, evaluated by ratio between MSC and MPC percentages, in control sub-cultures and “FLAT” conditions. Red and green areas indicate data from experiments that were considered valid (i.e., viability >80% and MSC/MPC <0.10 on controls). **(C)** Dot plots of representative sample included in the study. **(D)** Dot plots of representative samples excluded from the study for its consistent spontaneous differentiation in non-nanostrucutred controls.

**Figure 5:**
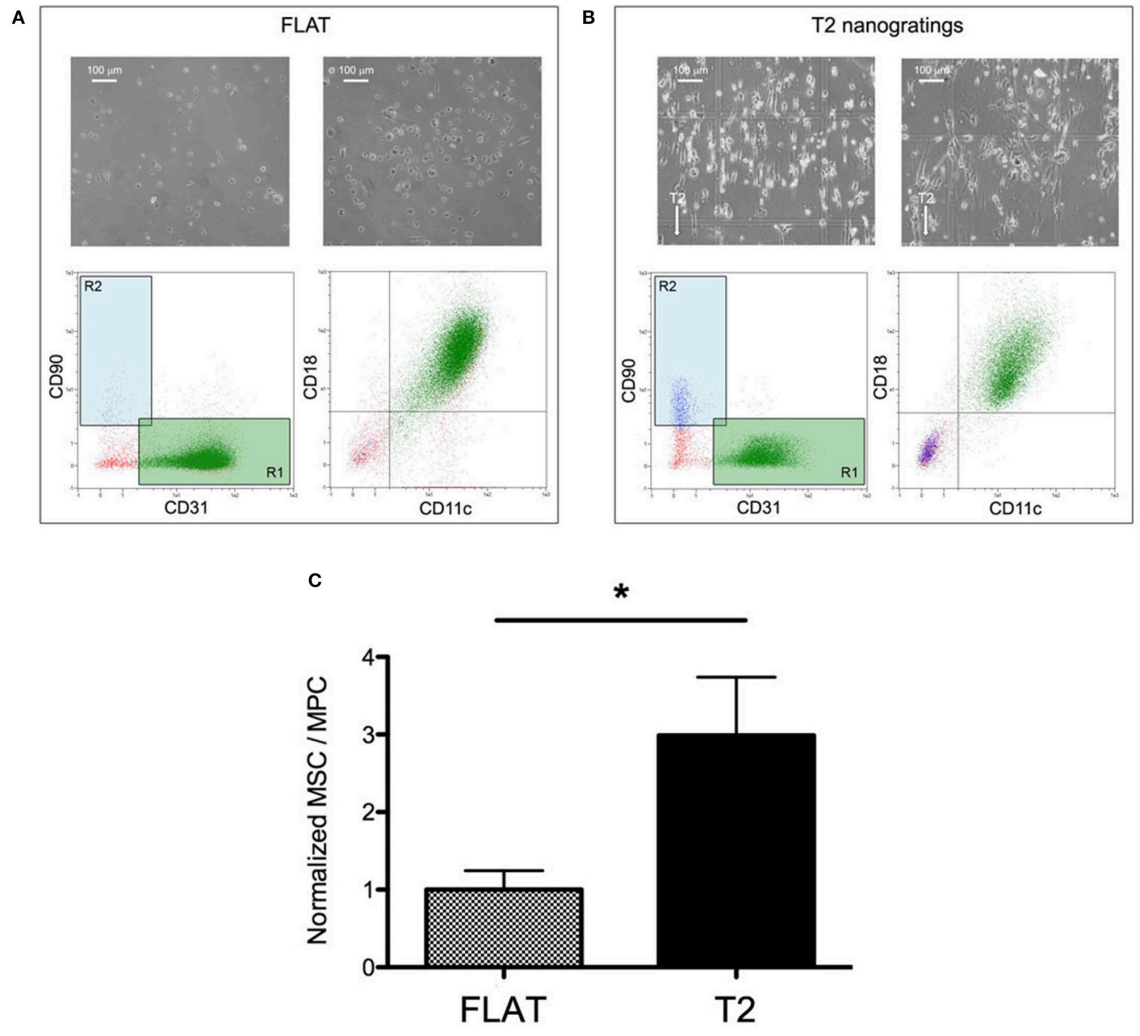
**Nanograting induced MPC-to-MSC differentiation**. Representative flow-cytometry scatter plots and microscopy images of MPCs cultured on Flat **(A)** and T2 **(B)**. **(C)** Ratio of viable MSCs over viable MPCs normalized to the average value measured on Flat (^*^p < 0.05, *t*-test).

In order to evaluate the effect of the solely topography of the T2 on the MPCs transition to MSCs, the cell mortally related to the re-plating procedures or to the different substrate chemical composition have been taken in consideration, eliminating the experiments that showed reduced vitality (<80%) in CTRL and in FLAT cultures (Figure 4A pale red area). For the same reason, possible spontaneous differentiation has been evaluated, and experiments in which CTRL or FLAT cultures showed a MSC/MPC ratio higher than 0.1 where excluded from the study (Figure 4B). After this evaluation eight experiments showed consistent vitality and absence of spontaneous differentiation (Figure 4C), while two experiments were excluded from the analysis (Figure 4D). After 7 days of culture on FLAT inserts, no change in MPC morphology or phenotype were reported and few events were detected in the *R2* gate, in all validated experiments (Figure 5A). Conversely on T2 NGs, evident signs of differentiation were proven by numerous elongated MSC-like cells oriented along the grating direction, and by a consistent population expressing MSC-associated phenotype, detected in the *R2* gate (Figure 5B). Quantitatively, normalized MSC/MPC ratio resulted significantly higher in cultures performed on T2 NGs (*p* < 0.05), with the mean value (0.19 ± 0.05) triplicated with respect to that measured on FLAT (0.062 ± 0.015, Figure 5C).

## Discussion

Implant osteointegration represents the key of success in most of the orthopedic procedures. It has been defined as the formation of structural and functional interconnection between the implant and the host bone (Albrektsson et al., 1981). In order to promote osteointegration, many materials and new fabrication processes have been proposed to obtain a high porous implant surface on which the bone cells could adhere and growth (Agarwal and García, 2015). Nowadays, the gold standard is represented by devices made by trabecular metal, which can mimic the bone microstructure and make available an ideal space for cellular colonization and revascularization of the neoformed bone tissue. However, an important limit of the implants and materials currently used in orthopedic surgery is that they present micron-sized textures while the bone is a nanostructured tissue. Collagen and hydroxyapatite, for example, provide a unique nanostructured scaffold for proteins and bone cell interactions. For these reasons, nanomaterials have been proposed with the aim of improving surface properties and creating an environment more conducive for osteoblast function and bone ingrowth (Tran and Webster, 2009; Parchi et al., 2013). Sub-micrometer sized features on traditional implants can control protein absorption and decrease scar tissue growth, bacterial infection and promote appropriate tissue growth. Moreover, they can also guide cell differentiation through physical stimulation, by a process called mechanotrasduction in which physical forces are converted into biochemical signals that are the finally integrated to give specific cellular response (Duncan and Turner, 1995).

Substrate nanotopography has been demonstrated to affect the biology of MSCs *in vitro* and the possibility of controlling the phenotype of these cells by just physically modifying the substrate topography represents one of the most promising innovations in the field. However, the majority of the studies was conducted with bone marrow derived mesenchymal stromal cells (BM-MSCs). Very recently our group demonstrated that nanogratings, alternate lines of sub-micrometer sized ridges and grooves, can effectively polarize BM-MSCs by pure contact interaction (Antonini et al., 2014), and promote enhanced osteo-differentiation with respect to flat surfaces (Antonini et al., 2016). There is indeed a large consensus considering BM-MSCs as the progenitors of the skeletal tissue related cell lineages for their ability to differentiate into adipocytes, osteoblasts and chondroblasts (Keating, 2012). This study also shows that after interaction with nanograting, BM-MSCs retain their osteodifferentiation potential.

Nonetheless, BM-MSCs represent a heterogeneous cell population isolated *in vitro*, in which the composition and cell biology are strongly affected by the isolating and expanding procedures (Phinney, 2012). Alongside the variability related to donors (Deasy et al., 2007; Siegel et al., 2013) and culture methods (Sharma et al., 2014), multiple origins of BM-MSCs were hypothesized and supported by several experiments demonstrating that genuine MSC cultures can be obtained by different BM subpopulations *in vivo* and *in vitro*, (reviewed in Pacini, 2014).

Remarkably, the MSC frequency in human normal bone marrow has been estimated only about the 0.001 and 0.01% of the mononuclear cells. Conversely, the MPCs frequency, and its *in vivo* counterpart denominated Pop#8, results from two to three logs higher (Trombi et al., 2009; Pacini et al., 2016).

Therefore, it is reasonable to hypothesize that the effects exerted by the nanostructured surfaces on MPCs can more strongly affect implant osteointegration than those on very small cell populations such us “skeletal stem cells” (SSCs) (Bianco and Robey, 2015) or CD271^+^CD140^low/−^ (Li et al., 2016), today considered the *bona fide* ancestors of BM-MSCs *in vivo*. Our results indicate that nanogratings can promote MPC to MSC transition, suggesting an *in vivo* scenario in which osteointegration is possibly promoted following MPC to MSC transition and nanograting-driven MSC osteogenic differentiation.

It is known that the substrate directionality stimulus is optimally delivered to many kind of undifferenziated cells, including MSCs, which in turn elongate and align to the nanograting lines. This polarization occurs also at level of cytoskeleton fibers and, though to a lesser extent, of nuclei. In this context, an elegant mechanistic model was proposed suggesting that an increased binding of integrins to ECM proteins would lead to increased FAK recruitment to the adhesion plaque inducing downstream ERK-dependent differentiation (Yee et al., 2008) This model provides an insight into the mechanisms of focal adhesion-dependent differentiation that might apply also to the MPC-MSC transition, and shows that nanotopographical surface modifications may directly regulate stem cell differentiation. Substrate topography can, indeed, interfere with focal-adhesion maturation and shaping, which in turn reflects on cellular mechanical stress distribution and shaping.

Interestingly, here we have demonstrated that the MPC mesengenic differentiation could be induced by the solely interaction with the T2 NGs, in absence of any other external stimuli. This aspect opens an interesting issue regarding the heterogeneity of culture expanded MSCs. In fact, it has been hypothesized that expanding MSCs from unfractionated “crude” bone marrow cell suspensions, in uncontrolled open culture systems, could lead to significantly different cell products with unpredictable biological properties because of mild modifications in the culture determinants or even to environmental fluctuations during cell expansion (Pacini, 2014). Data presented here suggest that differences in the intrinsic nanotopography of the culture surfaces, correlated to the materials or to the production processes, should be consider an additional culture determinant that might increase the variability of the final product. Thus, it might be possible that some pre-clinical data collected on MSCs expanded in flasks from bone marrow are not predictive for the therapeutic value of the same cell population cultured in bioreactors (i.e., equipped with hollow fibers), as their proliferation and differentiation potential could be altered by the specific micro- and/or nanotopography of the contact surfaces.

Concluding, the seminal findings reported here sustain the fascinating hypothesis of a topographic control of progenitor cells fate by specific designs of nanostructured surfaces. This aspect is of particular interest in the case of multipotent progenitors as MPCs that, retaining both mesengenic and angiogenic potential, possess a high clinical appeal.

## Author contributions

SA and MM: Conception and design, Data collection, assembly, analysis, and interpretation; EJ: Data collection, assembly, analysis, and interpretation; SM: Microfabrication of nanostrucutres; PP, SB, FP: Data collection, assembly, analysis, and interpretation; SP: Conception and design, Data collection, assembly, analysis, and interpretation. Manuscript writing; IP: Manuscript writing. MC: Conception and design, Data collection, assembly, analysis, and interpretation. Manuscript writing.

## Funding

This work was partially funded by “Centro per l'Uso Clinico delle Cellule Staminali” (CUCCS) as part of the project “Impiego di cellule stromali mesenchimali di origine midollare nelle pseudoatrosi, cisti ossee di astragalo e osteotomie in plus delle ossa lunghe” (project number 539999_2014_Petrini_CUCCS).

### Conflict of interest statement

The authors declare that the research was conducted in the absence of any commercial or financial relationships that could be construed as a potential conflict of interest.
